# Clinical risk prediction models for worsening heart failure events and all-cause mortality in adults with mild-to-moderate chronic kidney disease

**DOI:** 10.1093/eschf/xvaf037

**Published:** 2026-01-20

**Authors:** Soham Patel, Kaiwen Sun, Alan S Go, Michael P Girouard, Jane Y Liu, Rishi V Parikh, Thida C Tan, Emily S Lee, Rami Halaseh, Ankeet S Bhatt, Leonid Pravoverov, Sijie Zheng, Jana Svetlichnaya, Jesse K Fitzpatrick, Harshith R Avula, Keane K Lee, Sirtaz Adatya, David Ouyang, Parag Goyal, Alexander T Sandhu, Andrew P Ambrosy

**Affiliations:** Department of Medicine, Kaiser Permanente San Francisco Medical Center, San Francisco, CA 94115, USA; Department of Cardiology, Kaiser Permanente San Francisco Medical Center, San Francisco, CA 94115, USA; Division of Research, Kaiser Permanente Northern California, Pleasanton, CA 94588, USA; Departments of Medicine, Epidemiology, and Biostatistics, University of California, San Francisco, San Francisco, CA 94158, USA; Department of Cardiology, Kaiser Permanente San Francisco Medical Center, San Francisco, CA 94115, USA; Division of Research, Kaiser Permanente Northern California, Pleasanton, CA 94588, USA; Division of Research, Kaiser Permanente Northern California, Pleasanton, CA 94588, USA; Division of Research, Kaiser Permanente Northern California, Pleasanton, CA 94588, USA; Department of Cardiology, Kaiser Permanente San Francisco Medical Center, San Francisco, CA 94115, USA; Departments of Medicine, Epidemiology, and Biostatistics, University of California, San Francisco, San Francisco, CA 94158, USA; Department of Cardiology, Kaiser Permanente San Francisco Medical Center, San Francisco, CA 94115, USA; Division of Research, Kaiser Permanente Northern California, Pleasanton, CA 94588, USA; Department of Nephrology, Kaiser Permanente Oakland Medical Center, Oakland, CA 94611, USA; Department of Nephrology, Kaiser Permanente Oakland Medical Center, Oakland, CA 94611, USA; Department of Cardiology, Kaiser Permanente San Francisco Medical Center, San Francisco, CA 94115, USA; Department of Cardiology, Kaiser Permanente San Francisco Medical Center, San Francisco, CA 94115, USA; Department of Cardiology, Kaiser Permanente Walnut Creek Medical Center, Walnut Creek, CA 94596, USA; Division of Research, Kaiser Permanente Northern California, Pleasanton, CA 94588, USA; Department of Cardiology, Kaiser Permanente Santa Clara Medical Center, Santa Clara, CA 95051, USA; Department of Cardiology, Kaiser Permanente Santa Clara Medical Center, Santa Clara, CA 95051, USA; Division of Research, Kaiser Permanente Northern California, Pleasanton, CA 94588, USA; Department of Cardiology, Kaiser Permanente Santa Clara Medical Center, Santa Clara, CA 95051, USA; Department of Medicine, Weill Cornell Medicine, New York, NY 10021, USA; Division of Cardiology, Stanford University, Palo Alto, CA 94304, USA; Department of Cardiology, Kaiser Permanente San Francisco Medical Center, San Francisco, CA 94115, USA; Division of Research, Kaiser Permanente Northern California, Pleasanton, CA 94588, USA

**Keywords:** Heart failure, Chronic kidney disease, Predictive model, Outcomes, Morbidity

## Abstract

**Introduction:**

To develop and internally validate electronic health record (EHR)-based machine-learning models to predict worsening heart failure (WHF) events across care settings and all-cause mortality among adults with mild-to-moderate chronic kidney disease (CKD).

**Methods:**

We studied adults with mild-to-moderate CKD [estimated glomerular filtration rate (eGFR) 30–59 ml/min/1.73 m² or eGFR ≥60 with albuminuria] receiving care in a large health system from 2012 to 2021; outcomes were ascertained through 31 December 2022. Primary outcomes were (i) WHF events—outpatient encounters, emergency department (ED)/observation stays, and hospitalizations—identified using a validated natural language processing algorithm, and (ii) all-cause mortality. Models [extreme gradient boosting (XGBoost)] used an 80:20 train-test split and >500 EHR-derived covariates. Discrimination [area under the curve (AUC)] and calibration (slope) were evaluated overall and across subgroups by age, sex, race and ethnicity, and CKD stage.

**Results:**

Among 375 495 adults (mean age 64 ± 16 years; 54% women; 53% non-Hispanic White; mean eGFR 76 ± 26 ml/min/1.73 m²), the WHF model achieved AUC 0.887 (95% CI 0.879–0.893) with calibration slope 0.955; the mortality model achieved AUC 0.875 (95% CI 0.868–0.883) with calibration slope 0.914 in the test set. Performance was consistent across age, sex, and race and ethnicity, with a slight decrement as CKD stage worsened.

**Conclusions:**

Electronic health record-based machine-learning models accurately predicted WHF and mortality in mild-to-moderate CKD with strong calibration across key subgroups. These models are positioned for EHR deployment to support risk-stratified cardiovascular-kidney-metabolic care—prioritizing guideline-directed therapies and care pathways for those at highest risk.

## Graphical Abstract

**Graphical Abstract:**
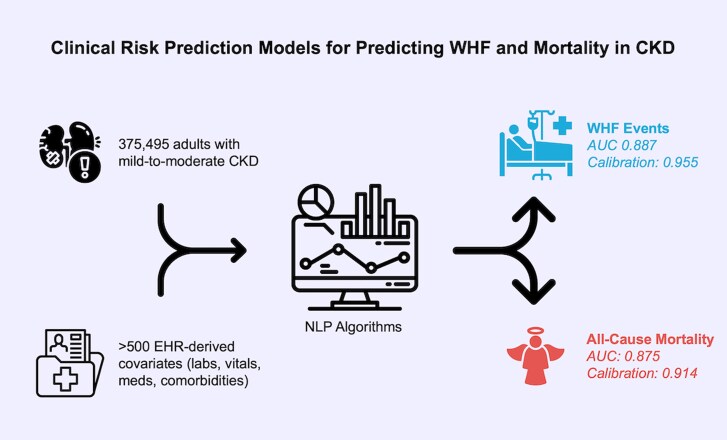
In a large cohort of adults with mild-to-moderate chronic kidney disease (CKD) receiving care in an integrated health system, we developed and internally validated electronic health record (EHR)-based machine-learning models using >500 routinely collected covariates and natural language processing (NLP)-identified worsening heart failure (WHF) events. The models demonstrated strong discrimination and calibration for predicting 1-year WHF events (AUC 0.887; calibration slope 0.955) and all-cause mortality (AUC 0.875; calibration slope 0.914), supporting scalable EHR deployment for risk-stratified cardiovascular-kidney-metabolic care pathways. Abbreviations: AUC, area under the receiver operating characteristic curve; CKD, chronic kidney disease; EHR, electronic health record; NLP, natural language processing; WHF, worsening heart failure.

## Introduction

Recognition of a unified cardiovascular-kidney-metabolic (CKM) syndrome is growing.^1^ Heart failure (HF) is a major driver of morbidity and mortality among people with chronic kidney disease (CKD), which affects >1 in 7 United States (US) adults.^2^ Conversely, even mild–moderate kidney impairment markedly elevates HF risk and up to 49% of patients with HF have renal dysfunction.^3^ Multiple therapy classes including sodium-glucose cotransporter-2 (SGLT2) inhibitors, glucagon-like peptide-1 receptor agonists, and non-steroidal mineralocorticoid receptor antagonists—reduce HF events, especially in diabetic or proteinuric CKD.^4–9^ Given the large at-risk population, accurate risk stratification is needed to implement a prevention framework that matches intervention intensity [e.g. guideline-directed medical therapy (GDMT) initiation and follow-up pathways] to individual risk.

Existing CKD-focused HF risk models, including those derived from the chronic renal insufficiency cohort (CRIC), exhibit suboptimal discrimination and calibration, thereby limiting clinical utility.^10–12^ Despite robust evidence that CKM-directed GDMT improves both renal and cardiovascular outcomes, substantial treatment gaps persist.^10,11^ We therefore applied advanced machine-learning methods to electronic health record (EHR) data to develop and validate scalable prediction models for worsening HF (WHF) events and all-cause mortality in adults with mild-to-moderate CKD. Our objective was to develop and internally validate EHR-based prediction models—robust to missing data and the challenges of real-world patient follow-up—to enable risk stratification for HF in CKD.

## Methods

### Setting and source population

Kaiser Permanente Northern California (KPNC) is a large, integrated healthcare delivery system that provides comprehensive care—including inpatient, emergency department (ED), and outpatient services—to over 4.5 million members through 21 hospitals and more than 260 clinics. The KPNC membership closely reflects the local and state-wide populations in terms of age, sex, race, ethnicity, and socioeconomic status.^13–15^ This study was approved by the KPNC Institutional Review Board, which also granted a waiver of informed consent given the retrospective design of the study.

### Study overview and cohort assembly

The study cohort comprised all adult (≥18 years) members of KPNC who showed evidence of mild-to-moderate CKD between 1 January 2012, and 31 December 2021. Mild-to-moderate CKD was defined as either: (i) an outpatient, non-ED estimated glomerular filtration rate (eGFR) between 30–59 ml/min/1.73m² for more than three months, based on the 2021 CKD-EPI race-free equation, or (ii) an outpatient, non-ED eGFR of ≥60 ml/min/1.73m² accompanied by laboratory evidence of proteinuria, indicated by a urine albumin-to-creatinine ratio ≥30 mg/g.^16,17^ Inclusion also required at least six months of continuous health plan enrolment with pharmacy benefits prior to the index date. Exclusion criteria included the presence of a left ventricular assist device, prior solid organ transplant, death on the index date, or lack of documented health plan membership following the index date.

### Follow-up and censoring

The index date was defined as the earliest date a participant met criteria for mild-to-moderate CKD. Baseline clinical and demographic data were ascertained from up to five years preceding the index date. Participants were followed from the index date until 31 December 2022, and they were censored at the earliest occurrence of health plan disenrollment, development of end-stage kidney disease (defined as initiation of maintenance dialysis or receipt of a kidney transplant), or death.

### Outcome ascertainment

WHF events and all-cause mortality were identified using validated natural language processing (NLP) algorithms applied to both structured and unstructured EHR data within the KPNC system. WHF events included hospitalizations (>24 h), ED or observation stays, and outpatient encounters (i.e. urgent care visits or visits with a primary care provider or cardiologist).

Worsening heart failure (WHF) events were defined using FDA-endorsed, standardized criteria requiring (i) a qualifying encounter, (ii) ≥ 1 HF symptom, (iii) ≥ 2 objective findings including ≥1 physical sign, and (iv) escalation of HF-directed therapy.^18^ These harmonized criteria were applied uniformly across outpatient, ED/observation, and inpatient encounters. Because WHF represents a continuum across care settings, we used a single internally validated NLP algorithm to identify events rather than developing separate setting-specific definitions or models, which would reduce model stability and add unnecessary complexity given lower event counts in some strata. Using a unified WHF definition across all care settings avoids artificially segmenting what is clinically a continuous decompensation spectrum and ensures model stability in strata with lower event frequency. This unified approach improves consistency, reflects real-world CKD care, and aligns with prior HF risk-prediction studies.

All-cause mortality was ascertained using a comprehensive, longitudinal mortality tracking system within the KPNC health system. Mortality status was derived from multiple, integrated data sources, including internal clinical records, hospital discharge abstracts, and linkage to external state and national death registries such as the California Death Statistical Master File and the Social Security Death Index.^14,19^ This multi-source integration ensures high completeness and reliability of vital status determination. Deaths were recorded regardless of cause or care setting (outpatient, emergency/observation, and inpatient) and were included in the analysis if they occurred during the observation period. The use of administrative, clinical, and registry-linked data enabled near-complete follow-up, thereby minimizing outcome misclassification.

The rule-based NLP algorithm was internally validated within the KPNC system against a gold standard of manual chart review, which included over 500 suspected WHF cases across care settings reviewed by two physicians, with final adjudication by a board-certified cardiologist in the event of discrepancies. The NLP algorithm has previously demonstrated excellent performance, with accuracy exceeding 90%–95% for identifying hospitalizations, ED visits or observation stays, and outpatient WHF encounters.^20,21^

### Data sources and covariates

Cardiac biomarkers such as N-terminal pro–B-type natriuretic peptide (NT-proBNP) and imaging metrics including LVEF were not routinely or uniformly available across encounters in this mild-to-moderate CKD population. To ensure that our prediction models remain deployable at scale across diverse clinical settings, we restricted predictors to routinely collected variables and excluded features with substantial systematic missingness. The >500 predictors therefore included raw EHR variables as well as derived longitudinal features such as variability, slopes, and encounter counts. XGBoost can internally accommodate correlated predictors without requiring pre-specification or exclusion for collinearity. Rather than enumerating each individual variable, predictors are described at the level of clinical domains, including demographics, comorbidities, medication exposures, laboratory measurements, vital signs, healthcare utilization patterns, and longitudinal trends derived from these inputs.

### Statistical analyses

We defined the person-year as the unit of analysis, allowing individuals with mild-to-moderate CKD to contribute repeated annual windows, each with distinct baseline and prediction intervals. Descriptive statistics were summarized using means with standard deviations for continuous variables and counts with percentages for categorical variables. Annualized WHF rates were calculated per 100 person-years, stratified by care setting, with 95% confidence intervals. Age- and sex-standardized rates were computed using the direct adjustment method. To account for the competing risk of death, we estimated cumulative incidence functions and used direct standardization to derive absolute risk estimates.^22–24^

Prediction models for WHF events (across all care settings) and all-cause mortality were developed using extreme gradient boosting (XGBoost), a tree-based machine-learning algorithm capable of handling nonlinear relationships, high-dimensional feature sets, and incomplete data. The dataset was randomly partitioned into training (80%) and testing (20%) subsets. Missing values were retained without imputation because XGBoost assigns missing observations to the optimal decision path during split finding, allowing for robust handling of routine EHR missingness. Hyperparameters were optimized through randomized grid search with five-fold cross-validation. All predictors—including raw variables and derived longitudinal features—were entered in their native units without transformation or normalization.

Model performance in the held-out test set was evaluated using multiple metrics. Discrimination was assessed using the area under the receiver operating characteristic curve (AUC) and the area under the precision-recall curve. Calibration was examined using calibration slope and mean squared error. We also report the maximum F1 score with its associated precision and recall. Confidence intervals for all performance metrics were estimated using 1000 bootstrap resamples. Variable importance was determined using the gain in predictive accuracy at tree-splitting nodes, and the top predictors for WHF and mortality were compared across models. All analyses were conducted using SAS version 9.4 and R version 4.3.1, with machine-learning workflows implemented using the XGBoost package.

## Results

### Cohort assembly and baseline characteristics

**Figure 1:**
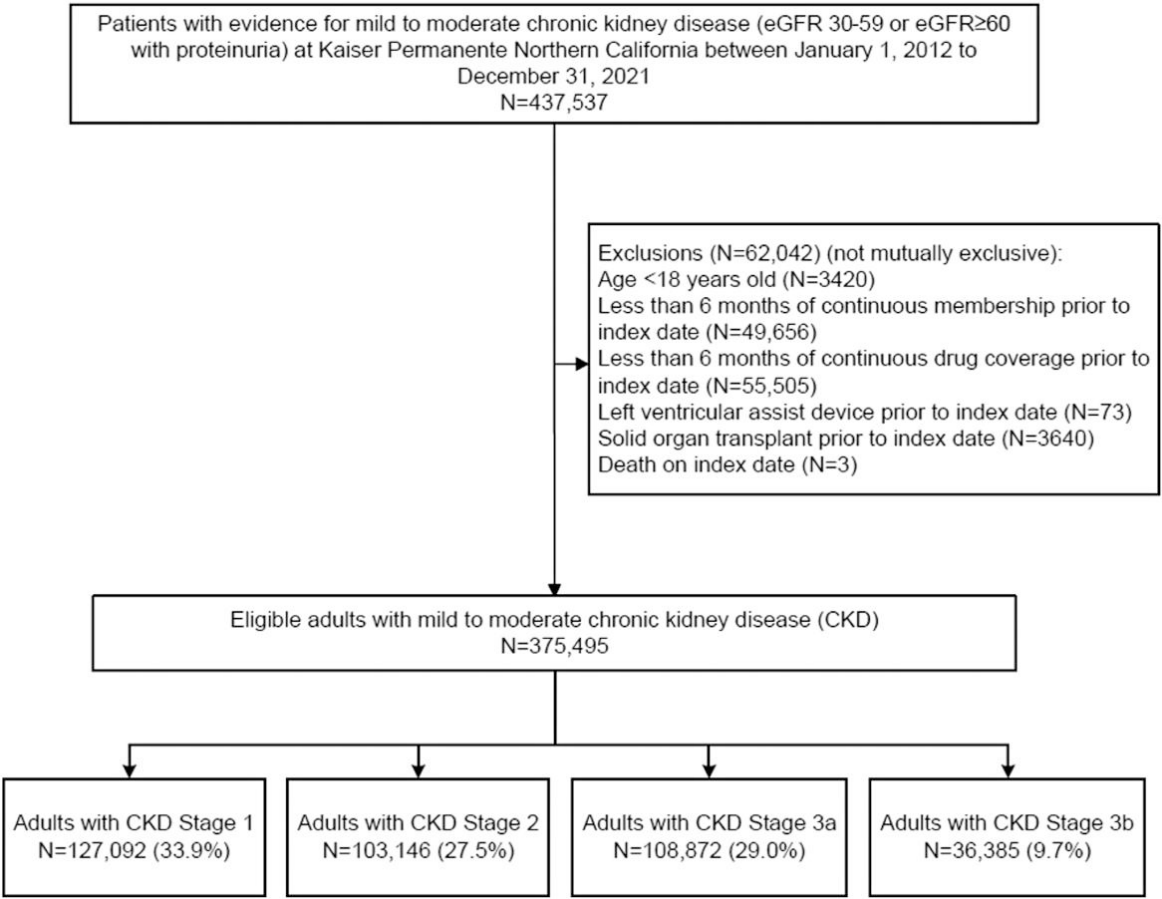
Study cohort assembly. Flow diagram of adults with mild-to-moderate chronic kidney disease in Kaiser Permanente Northern California (2012–21). Chronic kidney disease defined as estimated glomerular filtration rate 30–59 ml/min/1.73 m² or estimated glomerular filtration rate ≥ 60 ml/min/1.73 m² with proteinuria. Final cohort (*N* = 375 495) included chronic kidney disease Stage 1 (*n* = 127 092), Stage 2 (*n* = 103 146), Stage 3a (*n* = 108 872), and Stage 3b (*n* = 36 385). CKD, chronic kidney disease; eGFR, estimated glomerular filtration rate; LVAD, left ventricular assist device. Reproduced from the authors’ prior work, with permission.

We identified 375 495 adults with mild-to-moderate CKD between 2012 and 2021 for inclusion in the analytic cohort (*Figure 1*). The mean age was 64 ± 16 years, 54.2% were women, and 43% non-white (*Table 1*). The mean baseline eGFR was 76 ± 26 ml/min/1.73 m², and patients were predominantly classified into KDIGO categories G1A2, G3aA1, and G2A2. Loop diuretic use was present in 10.5% of patients. At baseline, 6.5% had HF and 8.7% had atrial fibrillation or flutter.

**Table 1:** Baseline characteristics of adults with mild-to-moderate chronic kidney disease between 2012 and 2021, overall and stratified by chronic kidney disease stage

Characteristic	Overall *N* = 375 495	CKD Stage 1 *N* = 127 092	CKD Stage 2 *N* = 103 146	CKD Stage 3a *N* = 108 872	CKD Stage 3b *N* = 36 385
**Age, years, *n* (%)**					
18–49	72 836 (19.4)	59 612 (46.9)	9402 (9.1)	2787 (2.6)	1035 (2.8)
50–59	59 791 (15.9)	30 892 (24.3)	17 672 (17.1)	8949 (8.2)	2278 (6.3)
60–69	91 491 (24.4)	25 699 (20.2)	32 107 (31.1)	26 946 (24.8)	6739 (18.5)
70–79	88 149 (23.5)	10 171 (8.0)	29 370 (28.5)	37 403 (34.4)	11 205 (30.8)
≥80	63 228 (16.8)	718 (0.6)	14 595 (14.1)	32 787 (30.1)	15 128 (41.6)
**Gender, *n* (%)**					
Male	171 956 (45.8)	54 715 (43.1)	56 012 (54.3)	46 450 (42.7)	14 779 (40.6)
Female	203 539 (54.2)	72 377 (56.9)	47 134 (45.7)	62 422 (57.3)	21 606 (59.4)
**Race/ethnicity, *n* (%)**					
Non-Hispanic White	176 124 (46.9)	40 999 (32.3)	46 331 (44.9)	66 929 (61.5)	21 865 (60.1)
Non-Hispanic Black	25 331 (6.7)	6599 (5.2)	7818 (7.6)	8184 (7.5)	2730 (7.5)
Hispanic	53 621 (14.3)	31 369 (24.7)	12 467 (12.1)	7088 (6.5)	2697 (7.4)
Asian	52 869 (14.1)	26 886 (21.2)	15 696 (15.2)	7826 (7.2)	2461 (6.8)
Native Hawaiian or Pacific Islander	2974 (0.8)	1707 (1.3)	874 (0.8)	291 (0.3)	102 (0.3)
American Indian or Alaska Native	1573 (0.4)	722 (0.6)	454 (0.4)	298 (0.3)	99 (0.3)
Multiracial	59 215 (15.8)	16 421 (12.9)	18 556 (18.0)	17 875 (16.4)	6363 (17.5)
Unknown	3788 (1.0)	2389 (1.9)	950 (0.9)	381 (0.3)	68 (0.2)
**Smoker, *n* (%)**					
Current	27 607 (7.4)	11 441 (9.0)	7762 (7.5)	6390 (5.9)	2014 (5.5)
Former	122 591 (32.6)	30 551 (24.0)	36 576 (35.5)	40 883 (37.6)	14 581 (40.1)
Never	225 297 (60.0)	85 100 (67.0)	58 808 (57.0)	61 599 (56.6)	19 790 (54.4)
**Medical history, *n* (%)**					
Heart failure	24 277 (6.5)	2276 (1.8)	7545 (7.3)	8808 (8.1)	5648 (15.5)
Atrial fibrillation or flutter	32 639 (8.7)	3496 (2.8)	10 966 (10.6)	12 632 (11.6)	5545 (15.2)
Ventricular fibrillation or tachycardia	1407 (0.4)	203 (0.2)	446 (0.4)	523 (0.5)	235 (0.6)
Ischaemic stroke or transient ischaemic attack	10 413 (2.8)	1559 (1.2)	3304 (3.2)	3855 (3.5)	1695 (4.7)
Acute myocardial infarction	7656 (2.0)	1134 (0.9)	2310 (2.2)	2808 (2.6)	1404 (3.9)
Mitral or aortic valvular disease	15 425 (4.1)	1620 (1.3)	4845 (4.7)	6205 (5.7)	2755 (7.6)
Venous thromboembolism	7985 (2.1)	1774 (1.4)	2647 (2.6)	2486 (2.3)	1078 (3.0)
Diabetes mellitus	91 133 (24.3)	46 071 (36.3)	29 178 (28.3)	11 766 (10.8)	4118 (11.3)
Hypertension	277 563 (73.9)	69 582 (54.7)	85 832 (83.2)	89 030 (81.8)	33 119 (91.0)
Dyslipidaemia	283 970 (75.6)	81 936 (64.5)	88 681 (86.0)	83 666 (76.8)	29 687 (81.6)
Hyperthyroidism	13 834 (3.7)	3308 (2.6)	3465 (3.4)	5174 (4.8)	1887 (5.2)
Hypothyroidism	55 730 (14.8)	11 592 (9.1)	15 093 (14.6)	20 989 (19.3)	8056 (22.1)
Chronic liver disease	22 523 (6.0)	10 441 (8.2)	7590 (7.4)	3457 (3.2)	1035 (2.8)
Chronic lung disease	98 386 (26.2)	28 022 (22.0)	28 815 (27.9)	30 307 (27.8)	11 242 (30.9)
Depression	55 100 (14.7)	15 836 (12.5)	15 955 (15.5)	17 408 (16.0)	5901 (16.2)
Dementia	10 412 (2.8)	778 (0.6)	2885 (2.8)	4563 (4.2)	2186 (6.0)
**Vital signs**					
BMI, kg/m^2^, mean (SD)	30.7 (7.3)	32.1 (7.9)	31.1 (7.2)	29.2 (6.4)	29.4 (6.7)
Missing	13 252 (3.5)	6206 (4.9)	4053 (3.9)	2291 (2.1)	702 (1.9)
Systolic blood pressure, mmHg, mean (SD)	130.8 (17.1)	129.6 (16.2)	132.0 (17.2)	130.8 (17.4)	131.6 (18.7)
Missing	6928 (1.8)	3530 (2.8)	2041 (2.0)	1050 (1.0)	307 (0.8)
**Laboratory values**					
Haemoglobin, g/dL, mean (SD)	13.5 (1.7)	13.7 (1.7)	13.6 (1.7)	13.4 (1.6)	12.7 (1.7)
Missing	58 097 (15.5)	22 705 (17.9)	17 135 (16.6)	14 589 (13.4)	3668 (10.1)
Estimated glomerular filtration rate, ml/min/1.73m^2^, mean (SD)	76.2 (26.1)	107.2 (11.9)	75.0 (8.8)	53.8 (4.2)	38.7 (4.3)
uACR, mg/g, mean (SD)	142.0 (672.9)	152.2 (641.9)	191.6 (905.0)	51.2 (204.8)	109.3 (352.7)
Missing	59 114 (15.7)	0 (0.0)	0 (0.0)	47 081 (43.2)	12 033 (33.1)
**Medications, *n* (%)**					
Angiotensin-converting enzyme inhibitor	136 245 (36.3)	36 108 (28.4)	42 063 (40.8)	42 792 (39.3)	15 282 (42.0)
Angiotensin II receptor blocker	70 803 (18.9)	18 553 (14.6)	25 915 (25.1)	19 422 (17.8)	6913 (19.0)
Angiotensin-neprilysin inhibitor	259 (0.1)	51 (0.0)	161 (0.2)	39 (0.0)	8 (0.0)
Mineralocorticoid receptor antagonist	7240 (1.9)	996 (0.8)	2297 (2.2)	2724 (2.5)	1223 (3.4)
Loop diuretics	39 432 (10.5)	4566 (3.6)	11 806 (11.4)	14 019 (12.9)	9041 (24.8)
Thiazide diuretics	103 569 (27.6)	21 593 (17.0)	29 236 (28.3)	39 568 (36.3)	13 172 (36.2)
β-Blocker	132 950 (35.4)	24 051 (18.9)	41 336 (40.1)	47 538 (43.7)	20 025 (55.0)
Calcium channel blocker	89 554 (23.8)	18 705 (14.7)	29 894 (29.0)	27 856 (25.6)	13 099 (36.0)
Antiarrhythmic drug	6724 (1.8)	796 (0.6)	1856 (1.8)	2819 (2.6)	1253 (3.4)
Oral anticoagulant	27 362 (7.3)	3770 (3.0)	9688 (9.4)	9776 (9.0)	4128 (11.3)
Antiplatelet drug	13 851 (3.7)	1982 (1.6)	4337 (4.2)	5057 (4.6)	2475 (6.8)
Statins	210 448 (56.0)	56 577 (44.5)	69 856 (67.7)	60 880 (55.9)	23 135 (63.6)
Other lipid-lowering drugs	15 157 (4.0)	3354 (2.6)	4641 (4.5)	4836 (4.4)	2326 (6.4)
Nitrates	13 647 (3.6)	1568 (1.2)	4116 (4.0)	4877 (4.5)	3086 (8.5)
Vasodilators	22 029 (5.9)	2600 (2.0)	6930 (6.7)	7431 (6.8)	5068 (13.9)
Any diabetes therapy	165 607 (44.1)	69 482 (54.7)	60 807 (59.0)	24 255 (22.3)	11 063 (30.4)
Sodium-glucose cotransporter 2 inhibitors	1808 (0.5)	834 (0.7)	892 (0.9)	69 (0.1)	13 (0.0)

Values are presented as *n* (%) or mean (SD) unless otherwise indicated. Missing data are shown where applicable. CKD stage based on KDIGO definitions: Stage 1 = eGFR ≥90 mL/min/1.73 m² with proteinuria; Stage 2 = eGFR 60–89 ml/min/1.73 m² with proteinuria; Stage 3a = eGFR 45–59 ml/min/1.73 m²; Stage 3b = eGFR 30–44 ml/min/1.73 m².

BMI, body mass index; CKD, chronic kidney disease; eGFR, estimated glomerular filtration rate; LVAD, left ventricular assist device; *n*, number; SD, standard deviation; uACR, urine albumin-to-creatinine ratio.

### Model performance for worsening heart failure

**Figure 2:**
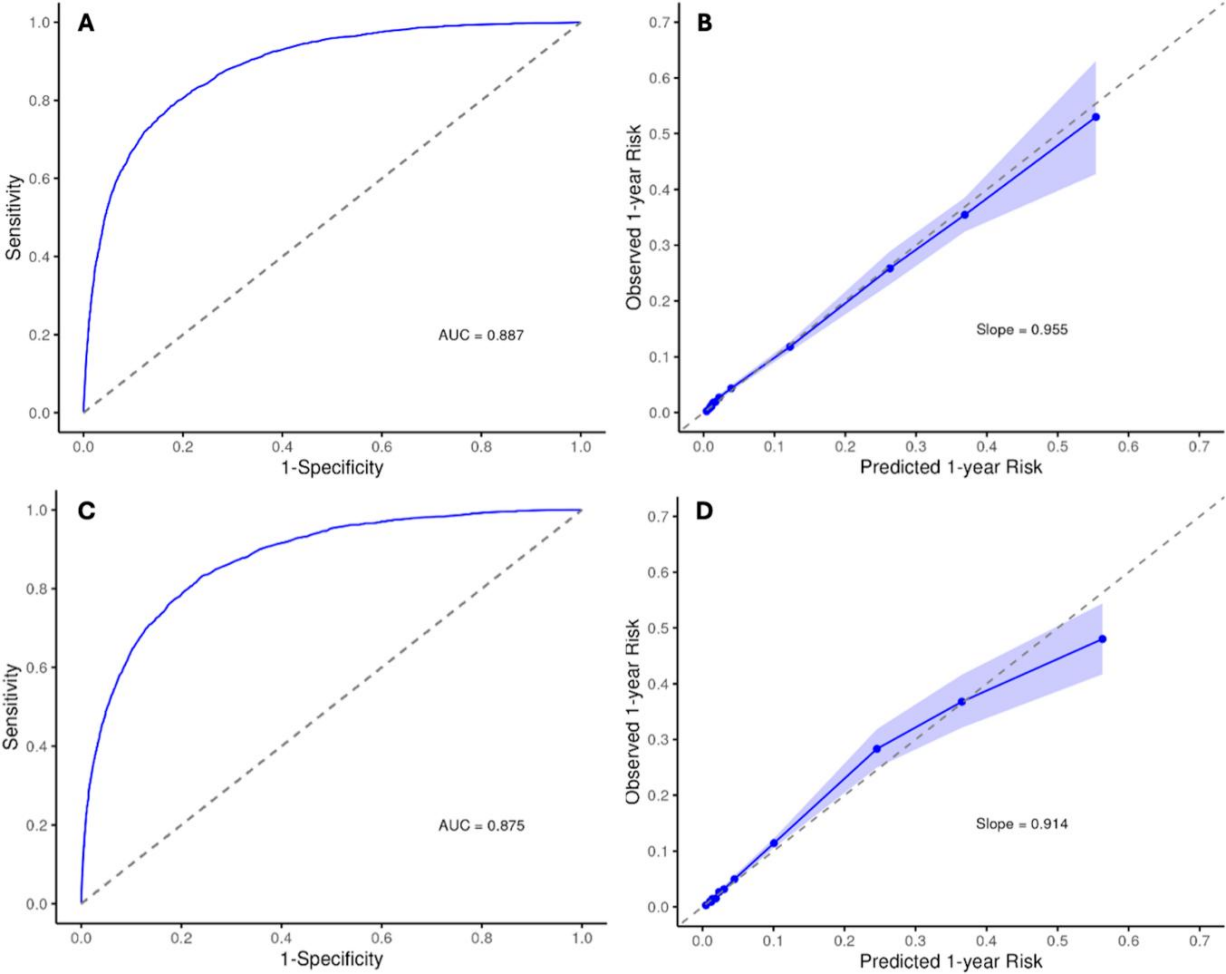
Discrimination and calibration of prediction models for worsening heart failure and all-cause mortality. Receiver operating characteristic curves for worsening heart failure (*A*) and mortality (*B*) showing area under the curve of 0.887 and 0.875, respectively. Calibration plots for worsening heart failure (*C*) and mortality (*D*) show slopes of 0.955 and 0.914, indicating strong agreement between predicted and observed 1-year risk. WHF, worsening heart failure; ROC, receiver operating characteristic; AUC, area under the curve

A total of 42 535 individuals (11.3%) experienced at least one WHF event during follow-up, corresponding to an overall event rate of 2.42 per 100 person-years (95% CI: 2.40–2.44). The WHF prediction model showed excellent discrimination with an overall AUC of 0.887 (95% CI: 0.879–0.893) (*Figure 2A*). Calibration was strong (slope 0.955; *Figure 2B*), indicating good agreement between predicted and observed risk.

Model performance remained robust across demographic and clinical subgroups. Area under curves for WHF prediction were high across age strata, ranging from 0.893 in individuals aged 18–49 to 0.822 in those ≥80 years. Performance by sex was comparable (AUC 0.888 in females vs. 0.883 in males). Discrimination remained strong across racial and ethnic groups, with AUCs ranging from 0.848 (non-Hispanic Black) to 0.946 (Native Hawaiian or Pacific Islander). Stratification by CKD stage revealed slightly lower performance in more advanced disease, with AUCs of 0.901, 0.872, 0.869, and 0.830 for Stages 1 through 3b, respectively (*Table 2*).

**Table 2:** Discriminative performance and calibration error of machine-learning models for predicting worsening heart failure events and all-cause mortality, overall and by subgroup

Characteristic	Outcome			
	Any WHF Event		All-Cause Mortality	
	AUC	MSE	AUC	MSE
**Age, years**				
18–49	0.893	0.004	0.902	0.004
50–59	0.897	0.012	0.862	0.014
60–69	0.865	0.017	0.869	0.020
70–79	0.851	0.029	0.850	0.027
≥80	0.822	0.049	0.808	0.044
**Gender**				
Female	0.888	0.019	0.893	0.017
Male	0.883	0.025	0.854	0.027
**Race/ethnicity**				
Non-Hispanic White	0.874	0.028	0.855	0.028
Non-Hispanic Black	0.848	0.024	0.874	0.017
Hispanic	0.898	0.015	0.878	0.016
Asian	0.884	0.012	0.901	0.013
Native Hawaiian or Pacific Islander	0.946	0.009	0.724	0.010
American Indian or Alaska Native	0.889	0.025	0.971	0.011
Multiracial	0.907	0.020	0.893	0.020
**CKD (Overall)**	0.887	0.022	0.875	0.021
Stage 1	0.901	0.009	0.913	0.015
Stage 2	0.872	0.027	0.853	0.036
Stage 3a	0.869	0.024	0.839	0.013
Stage 3b	0.830	0.045	0.785	0.028

Subgroup AUC and MSE values reflect model performance for 1-year risk prediction within each category.

AUC, area under the receiver operating characteristic curve; MSE, mean squared error; CKD, chronic kidney disease; WHF, worsening heart failure.

### Model performance for all-cause mortality

During the study, 24 468 deaths occurred. The mortality prediction model showed good discrimination, with an overall AUC of 0.875 (95% CI, 0.868–0.883) (*Figure 2C*). Calibration was strong (slope 0.914; *Figure 2D*), indicating close concordance between observed and predicted 1-year mortality.

Model performance remained consistently high across subgroups. Area under curves for all-cause mortality were >0.85 in nearly all age groups, though modestly attenuated with advancing age (AUC 0.902 for age 18–49 vs. 0.808 for age ≥80). Female patients had similar discrimination (AUC 0.893) compared to males (AUC 0.854). Performance was strong across race and ethnicity, with AUCs ranging from 0.724 (Native Hawaiian or Pacific Islander) to 0.971 (American Indian or Alaska Native). Performance also varied by CKD stage, with declining AUCs in more advanced disease: 0.913 for stage 1, 0.853 for Stage 2, 0.839 for Stage 3a, and 0.785 for Stage 3b (*Table 2*).

### Variable importance for worsening heart failure events and mortality

**Figure 3:**
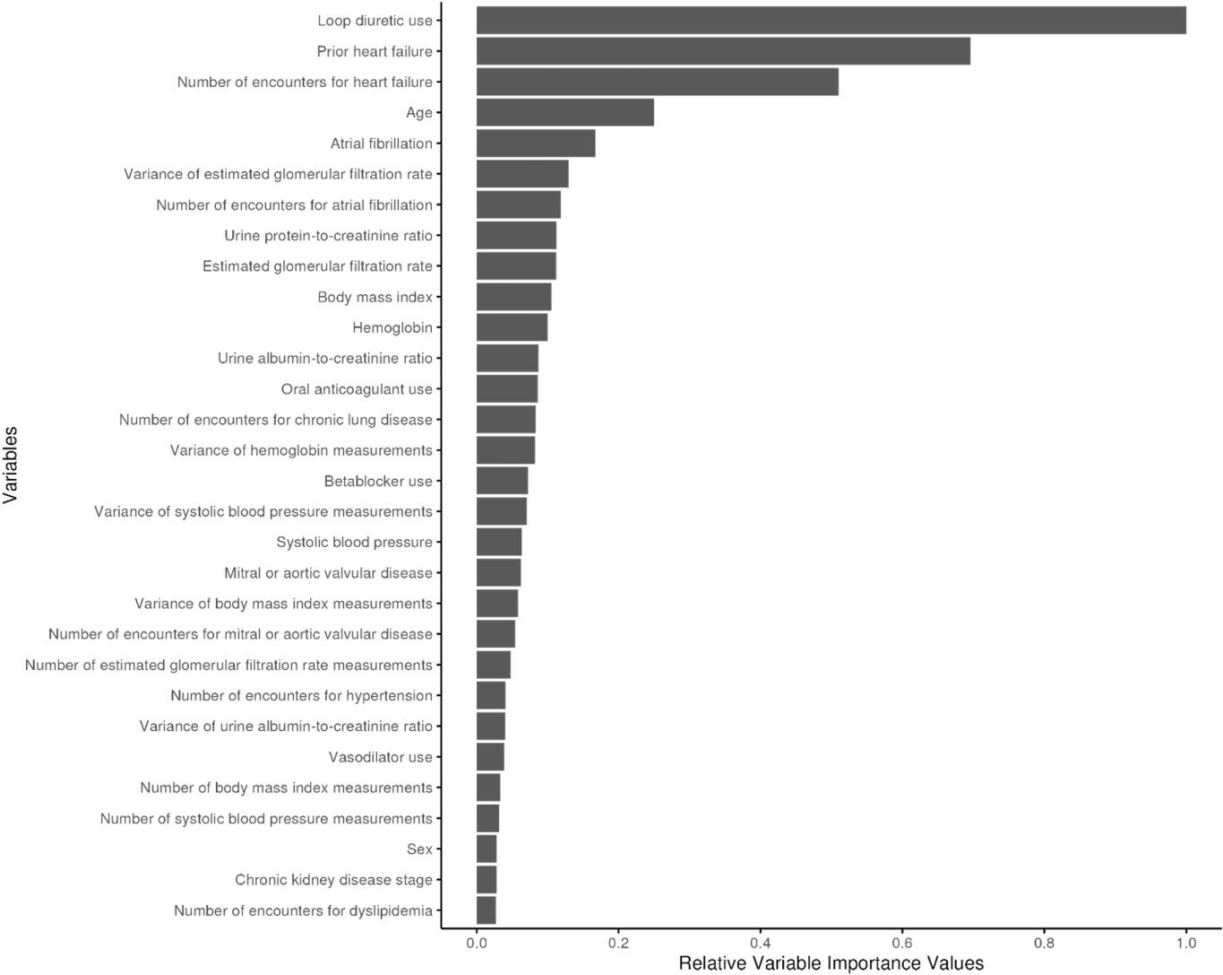
Relative variable importance for worsening heart failure events. Top predictors include loop diuretic use, prior heart failure, number of heart failure encounters, age, atrial fibrillation, and kidney function measures (estimated glomerular filtration rate, variability in estimated glomerular filtration rate, proteinuria). WHF, worsening heart failure; HF, heart failure; eGFR, estimated glomerular filtration rate

**Figure 4:**
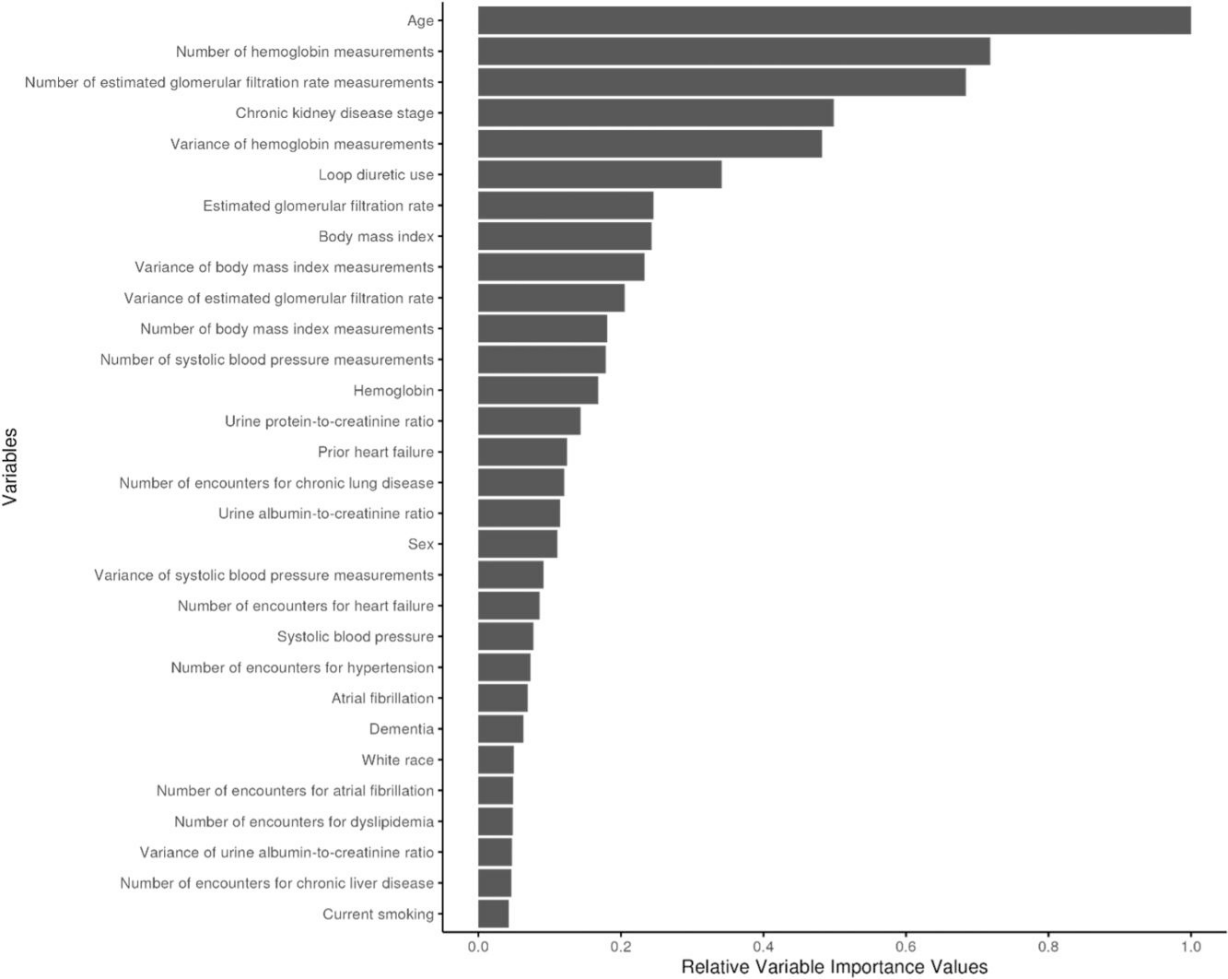
Relative variable importance for all-cause mortality. Top predictors include age, number of haemoglobin and estimated glomerular filtration rate measurements, chronic kidney disease stage, variability in haemoglobin, and loop diuretic use. CKD, chronic kidney disease; eGFR, estimated glomerular filtration rate

We analysed over 500 covariates—including geometric transformations, longitudinal summaries (e.g. counts, slopes, variances), and interactions from sociodemographics, comorbidities, vitals, labs, diagnostics, and medications—and identified the top 30 key predictors for each outcome. For WHF, the most influential variables included loop diuretic use, prior HF diagnosis, number of HF encounters, age, and atrial fibrillation, followed by kidney function measures such as eGFR variability and urine protein-to-creatinine ratio (*Figure 3*). Mortality prediction was driven primarily by age, intensity of laboratory surveillance (e.g. counts of haemoglobin and eGFR measurements), CKD stage, and haemoglobin variability (*Figure 4*).

## Discussion

In a large, diverse cohort of adults with mild-to-moderate CKD, we developed and internally validated EHR-based machine-learning models that accurately predicted WHF events and all-cause mortality using only routinely collected clinical data. Discrimination and calibration were strong and consistent across age, sex, race/ethnicity, and CKD stage, supporting broad applicability. Prior work shows that KPNC membership is broadly representative of surrounding US populations, enhancing generalizability.^13–15^ The models captured risk across the continuum of care, not solely during hospitalizations, and variable-importance patterns aligned with clinical expectations while leveraging high-dimensional EHR features that traditional scores often overlook.^3,12,19,25^

Predicting WHF in established HF is notoriously difficult due to clinical complexity and competing comorbidities,^26^ whereas incident WHF in CKD arises against a clearer baseline with well-characterized physiologic trajectories.^10–12,17,19^ This distinction likely explains the strong performance observed and highlights that EHR-based models may be especially valuable for early, risk-targeted HF prevention in CKD.

Model performance remained consistently strong across demographic and clinical subgroups. Although subgroup-specific calibration plots were considered, several strata had low event counts that would yield unstable curves; therefore, we assessed fairness using quantitative subgroup calibration metrics (calibration slope and MSE) in *Table 2*, which provide more reliable evaluation under sparse-data conditions. These numeric metrics provide consistent fairness evaluation even in the presence of sparse strata where graphical calibration plots would be unstable. Accuracy declined modestly in advanced CKD, likely reflecting overlapping HF and CKD-related volume overload, diuretic resistance, and fluctuating medical therapy.^3,27^

Variable-importance analyses revealed distinct risk pathways across outcomes. WHF risk was primarily driven by loop diuretic use, prior HF, atrial fibrillation, and kidney function trajectories—underscoring the interplay between baseline congestion, cardiovascular history, and renal dysfunction.^27^ Mortality prediction was dominated by age, multimorbidity, and laboratory-surveillance intensity, consistent with global disease burden.^25^ High-dimensional EHR data therefore provide rich prognostic signals that can guide outcome-specific interventions (e.g. volume optimization for WHF prevention and multimorbidity management to reduce mortality).

Beyond risk estimation, these models have clear potential for clinical implementation within contemporary EHR environments. Individualized risk estimates could be embedded in clinician dashboards, incorporated into automated high-risk alerts, or linked to structured CKM pathways prompting GDMT initiation, titration, and earlier follow-up. These integration routes leverage existing EHR infrastructure and align with modern health system priorities for scalable, risk-guided CKM care. Although formal workflow simulation or pilot deployment was beyond the scope of this retrospective modelling study, these strategies provide a practical foundation for prospective implementation. Importantly, the 1-year prediction horizon aligns with typical outpatient CKD and primary-care follow-up cycles, enabling incorporation of these risk scores into routine visit planning, population-health registries, and care-gap dashboards.

Traditional HF and CKD risk scores such as CRIC and MAGGIC rely on biomarkers or imaging tests (e.g. NT-proBNP, LVEF) that are incompletely captured in real-world CKD care, making head-to-head comparisons infeasible and susceptible to spectrum bias. Furthermore, unlike most prior EHR-based HF models, our study incorporates outpatient, ED/observation, and inpatient WHF encounters using an NLP-validated, FDA-endorsed definition, yielding a more complete representation of decompensation patterns in CKD. The discrimination achieved by our WHF (AUC 0.887) and mortality (AUC 0.875) models is comparable to or exceeds performance reported by these legacy scores in similar populations. By relying exclusively on routinely collected EHR data, our models complement rather than replace biomarker-based tools and provide a scalable alternative for CKM risk stratification in diverse care settings.

Because algorithmic bias is an important concern in EHR-based modelling, we evaluated subgroup performance across age, sex, race/ethnicity, and CKD stage, noting consistent discrimination and calibration. The models draw from core clinical features rather than proxies for socioeconomic or structural inequities. Prospective deployment should incorporate ongoing monitoring of subgroup performance, surveillance for calibration drift, and periodic recalibration to maintain equitable performance over time. Additionally, by avoiding proxies for socioeconomic or structural disadvantage (e.g. deprivation indices), the predictor set was intentionally limited to routinely collected clinical variables to reduce structural sources of algorithmic bias.

### Limitations

Our study has limitations. Despite using a large, diverse data set, models were still developed and internally validated within a single integrated health system; external validation is needed to assess transportability and guide recalibration. Because many traditional HF and CKD risk scores (e.g. CRIC, MAGGIC) rely on biomarkers or imaging measures not routinely captured in real-world CKD care—such as NT-proBNP and left ventricular ejection fraction (LVEF)—direct head-to-head benchmarking would exclude large portions of the population and introduce spectrum bias. Nevertheless, the discrimination achieved by our WHF (AUC 0.887) and mortality (AUC 0.875) models is comparable to or exceeds performance reported for these legacy scores in similar populations. By relying solely on routinely available EHR data, our models complement rather than replace biomarker-based tools and provide a scalable approach to risk stratification in settings where specialized cardiac testing is incomplete or unavailable.

Chronic kidney disease classification followed KDIGO definitions using routine eGFR and albuminuria, which are subject to measurement variability and potential misclassification. Care received outside the network may be missed, which could lead to under-ascertainment despite prior evidence that most care occurs within the system. Variable-importance rankings are model- and sample-dependent and should be viewed as hypothesis-generating rather than causal. Finally, although performance was consistent across demographic and CKD subgroups, small strata and temporal changes in practice may produce calibration drift; prospective deployment with ongoing monitoring and periodic recalibration will be essential.

## Conclusions

In summary, in a large and diverse CKD population, our EHR-based machine-learning models demonstrated strong discrimination and calibration for predicting WHF events and all-cause mortality, with consistent performance across age, sex, race/ethnicity, and kidney function strata. These findings support a clinically actionable, risk-stratified CKM framework. Practical EHR implementation pathways include embedding individualized risk estimates within clinician dashboards, generating automated high-risk alerts, and linking risk tiers to structured CKM pathways for GDMT initiation, titration, and earlier follow-up. While workflow simulation or pilot deployment was beyond the scope of this retrospective analysis, these strategies are readily supported by existing EHR infrastructure and align with ongoing digital-health initiatives. Prospective deployment with monitoring and periodic recalibration can operationalize this approach at scale and help close persistent treatment gaps in CKD.

## Declarations

### Data Availability

The data underlying this article cannot be shared publicly due to privacy and institutional restrictions. De-identified data may be made available upon reasonable request to the corresponding author, subject to approval by Kaiser Permanente Northern California and applicable data use agreements.

### Pre-registered Clinical Trial Number

None supplied.
